# Failure of observing NeuroD1-induced microglia-to-neuron conversion in vitro is not attributed to the low NeuroD1 expression level

**DOI:** 10.1186/s13041-022-00912-z

**Published:** 2022-04-05

**Authors:** Yanxia Rao, Bo Peng

**Affiliations:** 1 Department of Neurosurgery, Jinshan Hospital, Institute for Translational Brain Research, State Key Laboratory of Medical Neurobiology, MOE Frontiers Center for Brain Science, Fudan University, Shanghai, 200032 China; 2 Co-Innovation Center of Neuroregeneration, Nantong University, 226001 Nantong, China

**Keywords:** NeuroD1, Microglia, Conversion, Reprogramming, Expression level, Viral leakage, Artifact

## Abstract

NeuroD1-induced microglia-to-neuron conversion is hotly debated. Recently, we published a paper in *Neuron* demonstrating that NeuroD1 cannot induce microglia-to-neuron cross-lineage conversion. In the same issue of *Neuron*, Matsuda et al., who observed the “NeuroD1-induced microglia-to-neuron conversion” phenotype, responded to our study. They claimed that we failed to observe NeuroD1-induced microglia-to-neuron conversion in vitro due to the low NeuroD1 expression efficiency in our experiment. They argued that the NeuroD1 upregulation in our study was around 200-fold (vs. control), whereas the upregulation in Nakashima lab was 3000-fold, 15 times higher than ours. In fact, this is not true. We compared the expression level from the original paper and found that our NeuroD1 expression level was comparable to that of Matsuda et al. (Neuron 101:472–485.e477, 2019), or even higher. Therefore, the failure of observing NeuroD1-induced microglia-to-neuron conversion cannot be attributable to the low expression level.

## Main text

Recently, two latest papers from us and Chun-Li Zhang at UTSW demonstrated that the NeuroD1 cannot induce the glia-to-neuron conversion by microglia [1] or astrocyte [2]. The previous “NeuroD1-induced glia-to-neuron conversion” is most likely attributed to the viral leakages and experimental artifacts. When we published out study in *Neuron*, the group, who observed the “NeuroD1-induced microglia-to-neuron conversion”, responded to our study in the same issue of *Neuron*. They claimed that the lentivirus-induced NeuroD1 upregulation in our hands was ~ 200-folds (vs. control), whereas the upregulation in Nakashima lab was 3000-fold [3], 15-fold higher than our experiments. Thus, the reason we failed to observe NeuroD1-induced microglia-to-neuron conversion in vitro might be attributed to the low NeuroD1 expression efficiency in our experiments [4].

Matsuda and Nakashima said: *“We therefore first compared ND1 mRNA expression levels between the experiments of Rao et al. and our experiments in vitro. In their study using CAG-ND1-T2A-tdTomato lentivirus*, ***the relative ND1 mRNA expression in ND1-transducedcells was 200-foldhigher than that of control at 2 days after virus infection*** *(Figure S1E in *Rao et al. [1]*). However, this level of upregulation may be insufficient to induce MtN conversion. …… When we reevaluated ND1 mRNA expression in our system at 2 dpt, the RPKM value calculated from our original RNA sequencing (RNA-seq) data *[3]* was about 3000, and* ***the ND1 expression level relative to that in control was also 3000*** *(****Figure S1A and S1B****).”* [4].

**Fig. 1:**
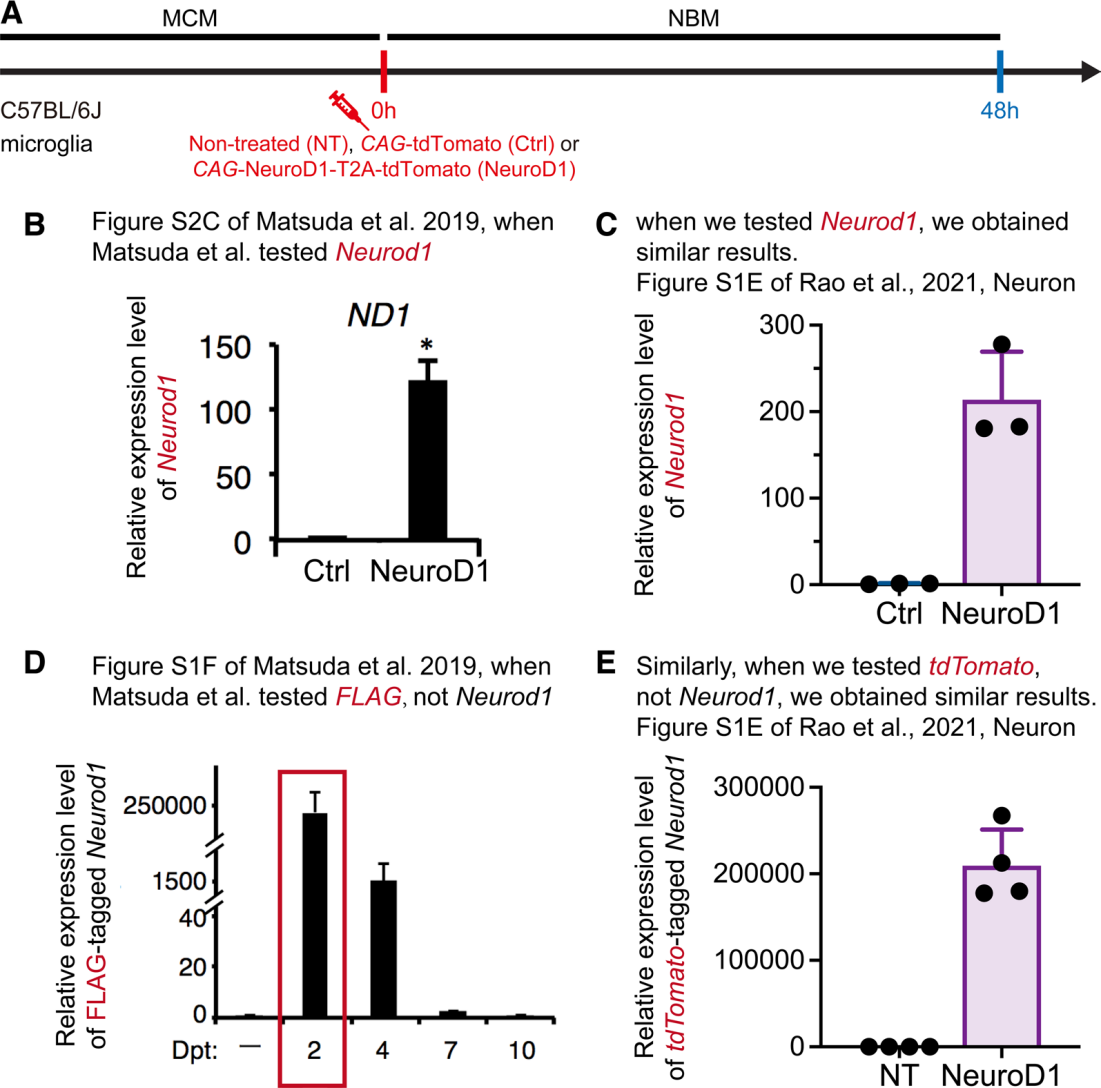
Comparison of the *Neurod1* and tag gene expression levels between Matsuda et al. [3] and Rao et al. [1]. **A** Scheme of in vitro lentiviral infection in primary microglial cell culture (for **C** and **E**). **B** qPCR results of *Neurod1* gene by Matsuda et al. [3]. **C** qPCR results of *Neurod1* gene by Rao et al. [1]. **D** qPCR results of *FLAG* gene by Matsuda et al. [3]. **E** qPCR results of *tdTomato* gene by Rao et al. [1]. *MCM* microglia culture medium, *NBM* neural basal medium, *NT* non-treated. Data are presented as mean ± SD. **B** and **D** are data from [3], **C** and **E** are data from [1], **B**, **C**, **D** and **E** are reproduced with permission from the authors

However, this is not true. The “3000-fold upregulation” by Matsuda et al. was obtained by a re-analysis from RNA-seq results [4], which was not shown in their original paper [3]. In contrast, the ~ 200-fold upregulation result in our study was acquired from qPCR [1]. The NeuroD1 expression levels cannot be compared under different methods and scenarios. As a matter of fact, Matsuda et al. did apply qPCR to evaluate the NeuroD1 expression in the primary cell culture system [3]. In their qPCR results, *Neurod1* exhibited a 125-fold upregulation at day 2 post lentiviral transduction (Fig. 1A, B), which is comparable to our qPCR results at day 2 post lentiviral transduction by the same method (Fig. 1 A, C; ~ 200-fold).

Moreover, Matsuda et al. also tested the expression of NeuroD1-linked reporter in their original paper. They observed an approximately 2,000,000-fold upregulation via qPCR (Fig. 1A, D) [3]. Notably, this is not the expression of *Neurod1*. Instead, it is the expression of the FLAG tag in their FLAG-tagged NeuroD1 vector design (NeuroD1-FLAG). Because this is a comparison to a non-treated control, which has no FLAG gene in nature, the fold-change (relative expression) could be very large (e.g. the expression level compares to a number approximately equal to zero). In our study, we also tested the relative expression level of the tdTomato tag after the lentiviral transduction of NeuroD1-tdTomato and compared to the non-treated control (Fig. 1A). We detected an approximately 200,000-fold upregulation of tdTomato tag expression (Fig. 1A, E) [1], comparable to the FLAG tag data of Matsuda et al. [3].

Therefore, the in vitro NeuroD1 expression level in our experiments was comparable to that of Matsuda et al., and probably our expression level was even higher (Fig. 1A–C, 120-fold of Matsuda et al. [3] vs. 200-fold of Rao et al. [1]). Obviously, the “low induction efficiency” cannot explain the failure of observing NeuroD1-induced microglia-to-neuron conversion in vitro. In contrast, our lineage tracing experiments have clearly demonstrated that the NeuroD1-induced “microglia-to-neuron conversion” is actually attributed to the viral leakage and experimental artifact.

Matsuda et al. argued that the expression DCX in their experiment was an evidence for appearance of immature neurons [3, 4]. However, we did not observe the *Dcx* expression in NeuroD1-transduced purified primary microglia (purify > 97%) [1]. Even though there are DCX expression induced by NeuroD1 in vivo, it cannot serve as an evidence for microglia-to-neuron conversion. Previous study demonstrated that DCX can be re-expressed in old mature neurons, which is termed as dematuration [5]. It is possible that the expression of DCX is a consequence of neuron dematuration upon NeuroD1-induction, especially in the leaky lentiviral system and without evidence from lineage tracing.

In our paper, we pointed out three generic principles for verifying the glia-to-neuron conversion. The most two important principles are: (1) the unambiguous glia-based lineage tracing, and (2) unambiguous live cell imaging (in vitro and/or in vivo) showing how an individual microglial cell converts to neuron. If NeuroD1 can indeed induce the microglia-to-neuron conversion, we respectfully suggest Matsuda and colleagues to provide convincing data showing the conversion, instead of arguing the low NeuroD1 dosage. Let alone the NeuroD1 expression levels are comparable, or even higher in our experiments. In addition, although the study of NeuroD1-induced microglia-to-neuron reprogramming [3] was cited by a lot of papers, none of them from a third party, unfortunately, successfully replicated the conversion.

## Data Availability

Not applicable.
